# Diabetes-associated *MYT1* and *ST18* genes regulate human beta cell insulin secretion and survival via other diabetes risk genes

**DOI:** 10.1007/s00125-026-06757-8

**Published:** 2026-06-05

**Authors:** Ruiying Hu, Nala Hamilton, Yu Wang, Xin Tong, Mahircan Yagan, Prasanna K. Dadi, Cristina Harmelink, Teri D. Doss, Jinhua Liu, Yanwen Xu, Alan J. Simmons, Ken S. Lau, Roland Stein, Appakalai N. Balamurugan, Irina Kaverina, Katie C. Coate, David A. Jacobson, Qi Liu, Guoqiang Gu

**Affiliations:** 1 Department of Cell and Developmental Biology, Vanderbilt University School of Medicine, Nashville, TN USA; 2 Department of Biostatistics and Center for Quantitative Sciences, Vanderbilt Medical Center, Nashville, TN USA; 3 Department of Molecular Physiology and Biophysics, Vanderbilt University School of Medicine, Nashville, TN USA; 4 Center for Computational Systems Biology, Epithelial Biology Center, Vanderbilt University School of Medicine, Nashville, TN USA; 5 Wendy Novak Diabetes Institute, Norton Children’s Research Institute, Norton Healthcare, Louisville, KY USA; 6 Department of Pediatrics, Division of Endocrinology, Pediatric Research Institute, University of Louisville, Louisville, KY USA; 7 Department of Medicine, Vanderbilt University Medical Center, Nashville, TN USA; 8 Department of Veterans Affairs, Tennessee Valley Healthcare System, Nashville, TN USA

**Keywords:** Beta cell apoptosis, Beta cell compensation, Beta cell dysfunction, Beta cell failure, Diabetes, Glucose-induced Ca^2+^ influx, Insulin secretion, Metabolic stress in beta cells, Mitochondria

## Abstract

**Aims/hypothesis:**

Genetic and environmental factors work together to cause islet beta cell failure, leading to type 2 diabetes. How these factors are integrated to regulate beta cells remains largely unclear. Based on our previous findings that the family of myelin transcription factors (MYT TFs; including MYT1, MYT1L and ST18) prevents mouse beta cell failure by repressing the overactivation of stress response, their regulation by obesity-related nutrition signals in human beta cells and their association with type 2 diabetes, we postulate that these factors prevent human beta cell failure under normal physiological conditions and obesity-related stress.

**Methods:**

*MYT1* or *ST18* were knocked down in primary human beta cells using shRNA. Beta cell survival, secretory function and gene expression were examined after islet cells were cultured in vitro or xenotransplanted into mice under normal conditions or obesity-related stress.

**Results:**

In culture, *MYT1-*knockdown (KD) caused beta cell death, while *ST18-*KD compromised glucose-stimulated insulin secretion. Under obesity-induced stress as xenotransplants, *ST18-*KD also caused beta cell death. Accordingly, *MYT1-*KD deregulated several genes and gene sets related to cell death and cellular stress response, while *ST18*-KD deregulated those regulating stress response, mitochondria and ion channels. Corresponding to these gene expression changes, *ST18*-KD reduced glucose-stimulated Ca^2+^ influx in beta cells. In addition, the *MYT1-* and *ST18*-regulated genes were enriched for type-2-diabetes-associated loci, with an enrichment of 2.05-fold relative to the random distribution of beta cell-expressed genes.

**Conclusions/interpretation:**

The MYT TFs complement each other to integrate genetic and environmental factors to prevent beta cell failure and type 2 diabetes, with their major effects exerted on beta cell viability and/or Ca^2+^ influx.

**Graphical Abstract:**

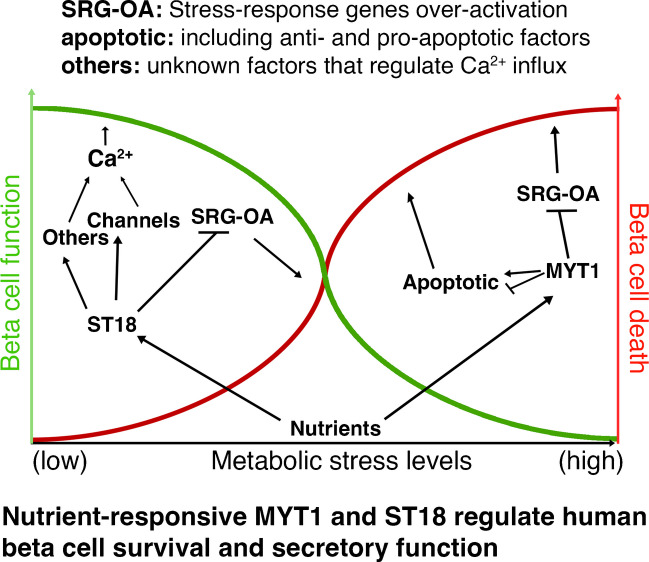

**Supplementary Information:**

The online version of this article (10.1007/s00125-026-06757-8) contains peer-reviewed but unedited supplementary material.


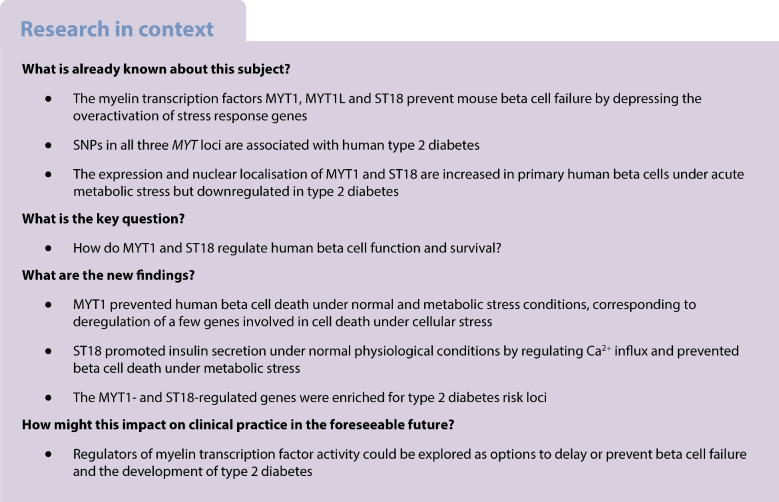


## Introduction

Type 2 diabetes develops when beta cells cannot secrete sufficient insulin to maintain whole-body glucose homeostasis. Hundreds of genetic loci have been associated with type 2 diabetes risk [1], most of which regulate beta cell dysfunction, loss of identity, or death (i.e. beta cell failure) [2, 3]. Environmental factors exacerbate the problem by overwhelming beta cells with excessive workload or accumulation of stressor molecules [4]. How beta cells integrate these factors to prevent this failure remains largely unknown.

A causative environmental factor for beta cell failure is obesity-induced insulin resistance [5–7]. Under this condition, beta cells increase insulin output in response to obesity-associated high glucose and NEFA levels. However, high levels of glucose metabolism, required for glucose-stimulated insulin secretion (GSIS), promote insulin biosynthesis. This co-produces unfolded proinsulin in the endoplasmic reticulum (ER) that, if not cleared, induces ER stress and dysfunction [8]. High glucose and NEFA metabolism in beta cells also produces reactive oxygen species and/or toxic lipid metabolites [9]. These products, at high levels, cause oxidative stress while also exacerbating ER stress. Henceforth, beta cells activate the stress response to maintain homeostasis [10, 11]. However, stress responses can persist too long or be overly activated, reducing essential beta cell proteins or inducing proapoptotic genes [5, 12]. Thus, precise regulation of the stress response is critical for preventing beta cell failure [13, 14].

The myelin transcription factor (MYT TF) family consists of three zinc finger proteins expressed in neuronal and endocrine cells (MYT1 [NZF2], MYT1L [MYT2, NZF1] and ST18 [MYT3, NZF387]) [15, 16]. In mice, these factors regulate beta cell function, survival and proliferation by repressing several stress response genes [17–21]. In humans, SNPs and deletions in these genes are associated with autism, cancer, craniofacial anomalies, diabetes or mental illnesses [1, 15, 22–26]. Intriguingly, the protein levels and/or nuclear localisation of MYT1 and ST18 are regulated by glucose and NEFA in primary human beta cells, and the reduced production of MYT TF precedes beta cell dysfunction during type 2 diabetes development [18].

The above studies suggest that the MYT TFs are environment-responsive regulators that prevent mouse beta cell failure. Our objective here is to investigate this possibility in primary human beta cells, focusing on *MYT1* and *ST18* because they, but not *MYT1L*, are regulated by high glucose or NEFA in human islets [18]. For this goal, transplantation of manipulated human islets into mouse models was used to mimic in vivo cell responses to obesity-related stress.

## Methods

### Human donor islet and mouse usage

Human donor islets, de-identified (exempt from Institutional Review Board review), were obtained from the IIDP or A. N. Balamurugan’s islet cell laboratory (University of Louisville, KY, USA) [27]. Islets with >80% purity, >95% viability and a stimulation index >2.0 were used (see electronic supplementary material [ESM]: Human islet checklist). Mouse usage was approved for G. Gu by the Vanderbilt University Institutional Animal Care and Use Committee, in accordance with the policies of the Office of Laboratory Animal Welfare. Human donor islets were procured whenever available to ensure randomisation. Mice used as transplantation recipients were randomly picked.

### Lentiviral vector production

We used HEK293 cell culture to identify *MYT1-*Si3 *shRNA* (5′-CCTCACCTAAAGCCTTTCAAT-3′) and *ST18-*Si4 *shRNA* (5′-GCCGAAATAGAAGTGGATGAA-3′) for *MYT1* or *ST18* knockdown (KD), respectively (ESM Fig. 1). Lentiviral vectors were made to express eGFP and shRNA for KD in human pseudo-islet (PSI) cells. See ESM Methods (HEK293 cell-culture-based reporter assays).

### qRT-PCR

RNA was isolated using DNA-free RNA kits (Zymo Research), converted to cDNA (Promega’s High-Capacity cDNA Synthesis Kit), and analysed with the Bio-Rad SYBR Green Master Mix (Bio-Rad Laboratories). Oligos were as follows: *GAPDH*, 5′-CTTTGGTATCGTGGAAGGACTC-3′ and 5′-AGTAGAGGCAGGGATGATGT-3′; *MYT1*, 5′-GGCCACATCACCGGGAACTA-3′ and 5′-AGTGGGCAGCCATGAGGTTT-3′; *MYT1L*, 5′-CAGCTGCTGCCATCCTGAAC-3′ and 5′-GCCCGCTGTTTGATGGTCAG-3′; and *ST18*, 5′-GGCATGCAGACTCTGTGGCT-3′ and 5′-CATCCACAGCCAGCCATTCG-3′.

### Pseudo-islet production, transplantation and insulin tolerance tests

For PSI production, dissociated islet cells were washed and resuspended in the Vanderbilt-Pseudo-islet-media (VPM) (50% CMRL1066 + 50% Vasculife Basal Media, 10% FBS, 1× antibiotics, 0.5× Glutamax, 2.5 mmol/l HEPES, 0.5× sodium pyruvate plus recombinant human VEGF, EGF, FGF basic, IGF-1, ascorbic acid, hydrocortisone hemisuccinate, heparin sulphate LifeFactorGentamicin/Amphotericin, and iCell endothelial cell medium supplement, all from R&D Systems). Cells (200,000 in 200 μl) were aliquoted into one prepared Aggrewells 96-well plate and mixed with 40–50 ng of lentivirus. The plate was centrifuged at 200 *g* for 5 min and left undisturbed in a cell-culture incubator for 5 days to allow PSI formation. Well preparation used 100 μl/well of an anti-adhesive rinsing solution (96-well plate; Nacalai 4860-900SP, Nacalai USA) [28].

Roughly 30 PSIs were transplanted into anterior eye chambers (AECs) of 2- to 4-month-old NSG-DTR mice (027976, the Jackson Laboratory) kept in small cages in a pathogen-free facility with a 12 h dark–light cycle without food/water restrictions [29]. Transplants were recovered 4 weeks after transplantation or after another 12 weeks on a control (~13% energy from fat, Lab Diet) or a high-fat diet (HFD, ~60% energy from fat, Fisher Scientific). For insulin tolerance tests, mice were fasted for 4 h, injected with Humalog insulin (0.5 U/kg), and blood glucose was tested at 0, 15, 30, 45 and 60 min after injection.

### TUNEL assays and immunofluorescence staining

TUNEL assays used kits from Invitrogen. Antibodies for immunofluorescence staining were as follows: rabbit or guinea pig anti-PDX1 (1:5000, or 1:2000) (gifts from C. Wright, Vanderbilt University); goat anti-somatostatin (1:500) (Millipore, RRID:AB_22555365); rabbit anti-somatostatin (1:2000) (Santa Cruz Biotechnology, RRID:AB_2195930); rabbit anti-pancreatic polypeptide (1:500, RRID:AB_3065109) and rabbit anti-TOMM20 (1:500, RRID:AB_3065091) (Abcam); guinea pig anti-insulin (1:1000, RRID:AB_10013624) and mouse anti-glucagon (1:5000, RRID:AB_2234057) (Dako); rabbit anti-MYT1 (1:1000) and rat anti-ST18 (1:1000) (in-house); rabbit anti-MafA (1:1000, Novus, RRID:AB_1503594); and chicken anti-NKX6.1 (1:500) (Developmental Studies Hybridoma Bank, F55A10). Validation used antigen-negative tissues/cells. Secondary antibodies were from Jackson ImmunoResearch (1:1000, catalogue no. available upon request). Slides were counterstained with DAPI, imaged with laser-scanning confocal microscopy or Airyscan (FV1000 or Leica 880), and quantified with ImageJ (National Institutes of Health, USA).

### Secretion assays

PSIs were pre-incubated in KRB (with 2.8 mmol/l glucose [G2.8]) at 37°C for 1 h, split into 3 or 4 wells (15–20 PSIs each), and stimulated with G2.8 then 16.7 mmol/l glucose (G16.7) for 45 min. PSIs were lysed with ethanol/HCl to obtain total insulin/glucagon amounts. KCl-induced secretion used 30 mmol/l KCl. ELISA kits from Alpco and Mercodia were used to quantify insulin and glucagon, respectively.

### RNA-seq, single-cell RNA-seq and function annotation

For RNA-seq, RNA preps with RIN 7.6 were sequenced on the Novaseq 6000, yielding 100–120 million reads per sample. The gene count matrix (60,683 total genes) was from Genialis [30]. Lowly expressed genes were excluded by requiring a minimum of three reads in at least three of the 12 samples, resulting in 34,266 genes retained for downstream analysis (ESM Table 1). Differentially expressed genes (DEGs) were identified using DESeq2 [31] with a paired design, and *p* values were adjusted using the Benjamini–Hochberg procedure. DEGs were defined by a log_2_ fold-change threshold of 0.5 and an FDR-adjusted *p* value cutoff of 0.05. Functional enrichment analysis was performed against Gene Ontology (GO) database with clusterProfiler [32]. Gene set enrichment analysis (GSEA) was conducted using the fgsea R package with 35,134 gene sets from MSigDB (version 2025.1.Hs. www.gsea-msigdb.org/gsea/index.jsp) [33, 34].

Single-cell RNA-seq (scRNA-seq) analyses followed routine methods to identify cells with high-quality data (see ESM Methods: scRNA-seq quality control for details). Reads were normalised using UMI-filtered counts. Cell subpopulations were identified and visualised using UMAP in Seurat, based on the first 30 principal components from the top 2000 highly variable genes [35, 36]. Seurat identified DEGs at the criteria of |log_2_ fold-change|>0.25 & FDR≤ 0.05. Pathway analysis used Database for Annotation, Visualization and Integrated Discovery (DAVID) for functional clustering [37] (*p*_adj_<0.05, Benjamini–Hochberg procedure), enabling functional clustering of similar processes for data interpretation. To identify the specific functions, each gene was searched for association with secretion, stress or cell death/apoptosis within the STRING database [38]. We then verified the annotation by examining published data.

### Mitochondria and insulin assays

To examine mitochondrial volume and insulin vesicle numbers in beta cells, cells were stained for TOMM20 and insulin, imaged and quantified. Mitochondrial transmembrane potential assays were done in dissociated islet cells incubated with MitoView 633 with flow cytometry (see ESM Methods: Mitochondrial assays for details).

### Ca^2+^ imaging

Dissociated islet cells attached to glass-bottomed plates (D35-14-1.5P, Cellvis) were infected with shRNA-expressing lentivirus for 3 days, and with RCaMP-expressing adenovirus (beta cell specific) overnight. Cells were then incubated (20 min, 37°C) in KRB with 1 mmol/l glucose (G1). Real-time fluorescence imaging (5 s per frame) followed, with a 20× objective, and recordings for 5 min under G1, 25 min under 11 mmol/l glucose (G11) and 5 min under 30 mmol/l KCl.

### Overlapping MYT-regulated and type-2-diabetes-associated genes

For PSI- or beta cell-specific gene overlapping assays by hypergeometric tests, we used the 34,266 PSI (ESM Table 1) or 18,742 beta cell genes (ESM Table 2; expressed in beta cells with TPM >0.5 [18,350] in [39] plus those extras from Walker et al [40] or our scRNA-seq analysis) as starting populations, respectively. Six type 2 diabetes gene lists were used: all type 2 diabetes-associated genes from the GWAS Catalog (*p*<9 × 10^−6^, [41]) that are expressed in PSIs (2047 genes; ESM Table 3) or beta cells (1806; ESM Table 4); type 2 diabetes genes from Suzuki et al (*p*<5 × 10^−8^) [1] that are expressed in human PSIs (665; ESM Table 5) or beta cells (621; ESM Table 6); and the DEGs between islets (686; ESM Table 7) or beta cells (365; ESM Table 8) from healthy donors vs donors with type 2 diabetes from the Walker studies [40].

### Statistical analysis

Double-blind sample characterisation/quantification methods were used whenever possible. Differences between two groups were assessed using the Mann–Whitney *U* test (independent samples) or the Wilcoxon signed-rank test (paired samples). A *p*<0.05 accompanied by consistent directionality across all donors was interpreted cautiously as suggestive evidence of statistical significance. Linear mixed-effects models (LMMs) were used for experiments with repeated measurements, either in designs with ten or more independent samples across multiple time points (six or more) or in nested designs (hundreds of cells per sample with repeated measurements over hundreds of time points), to account for within-sample and within-cell correlation, with statistical inference interpreted cautiously. Hypergeometric tests were used to assess whether the overlap between two gene sets was greater than expected by chance, given a defined background set of genes.

## Results

### High levels of *ST18* are required for human beta cell GSIS

**Fig. 1:**
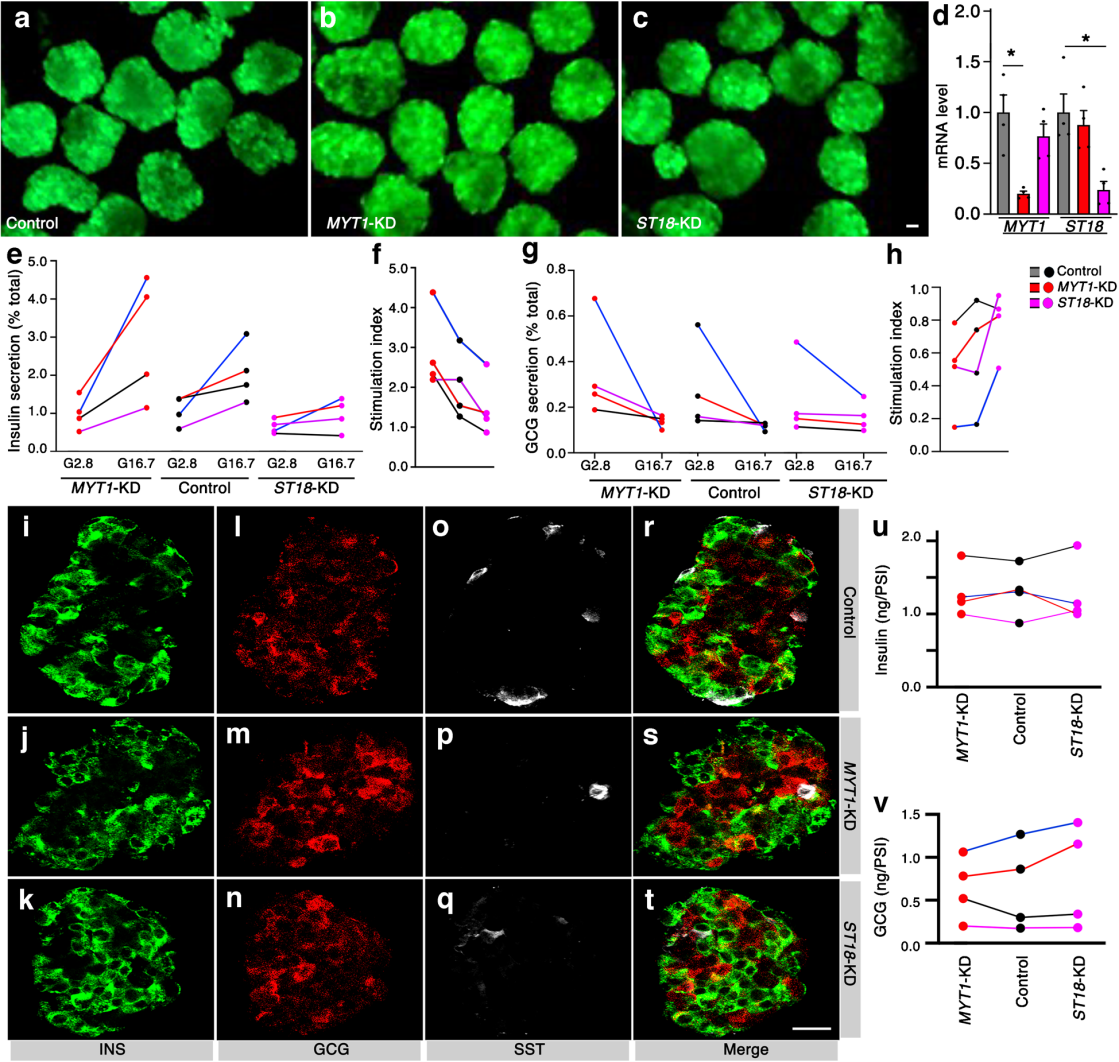
*ST18*-KD, but not *MYT1*-KD, impairs GSIS of primary human beta cells. Human islets, after quality check, were dissociated into single cells, infected with an eGFP-expressing lentivirus that also expressed control, *MYT1*-targeting or *ST18*-targeting shRNAs and then used to make PSIs and tested. (**a**–**c**) Representative PSI images. Scale bar, 20 μm. (**d**) qRT-PCR assays showing *MYT1*-KD or *ST18*-KD levels in PSIs made from four donors. The relative mRNA levels of *MYT1* and *ST18* are shown, with their expression under control shRNA set as 1 (template loading was normalised against *GAPDH*). The grey bars in the *MYT1* or *ST18* assay subgroups were shared controls for the *MYT1-*KD or *ST18-*KD samples. Each circle represents data from one donor. Data are shown as mean + SEM. *p=0.028 (analysed by Mann–Whitney *U* test). (**e**–**h**) PSI secretion of insulin (**e**, **f**) or glucagon (**g**, **h**) in response to G2.8 or G16.7, presented as % of total hormone secreted (**e**, **g**) or as stimulation index (**f**, **h**). Connected lines show islets from the same donor. Each circle represents the average of three or four technical replicates of the PSIs from one donor. The colour of the connecting lines indicates donor identity (black, blue, red and pink lines correspond to donors 1, 2, 3 and 4, respectively). In (**e**) and (**f**), all four donors showed consistent reduction in their insulin secretion (% total or stimulation indices;* p*=0.06 for both, analysed by Wilcoxon signed-rank tests) in response to G16.7. Sum of positive, negative ranks: 0.00, −10.00. No consistent changes were observed for *MYT1*-KD samples or for glucagon secretion in *ST18*-KD samples. (**i**–**t**) Hormone expression in newly produced PSIs, represented by samples from donor 1. Single channels (**i**–**q**) and merged panels (**r**–**t**) are shown. Scale bar, 20 μm. (**u**, **v**) Insulin (**u**) and glucagon (**v**) content in PSIs after *MYT1*-KD or *ST18*-KD, normalised against the numbers of PSIs of similar size. Each circle represents the average of three or four technical replicates from a single donor. Connected circles are from the same donor. The colour of the connecting lines indicates donor identity (black, blue, red and pink lines correspond to donor 1, 2, 3 and 4, respectively). No consistent changes in gene expression were seen. GCG, glucagon; INS, insulin; SST, somatostatin

Lentiviral vectors producing *MYT1-Si3* or *ST18-Si4* shRNAs reduced the *MYT1* and *ST18* mRNA levels by 79.9 ± 2.5% (mean ± SEM) and 74.2 ± 8.3%, respectively (both *p*<0.05), in human PSIs (Fig. 1a–d). *MYT1*-KD did not change either the percentage of insulin secreted under stimulation with G16.7 or the stimulation index (secretion at G16.7/stimulation at G2.8) (Fig. 1e, f). In contrast, *ST18*-KD consistently reduced PSI GSIS compared with controls in all four donor islet batches tested when the percentages of total insulin secreted (52.0 ± 9.1% reduction) or when the stimulation index (26.7 ± 7.2% reduction) were examined (Fig. 1e, f), although the Wilcoxon signed-rank test did not produce statistically significant *p* values (*p*=0.06).

There was no difference in glucagon secretion in control, *MYT1*-KD and *ST18*-KD PSIs (Fig. 1g, h). *MYT1*-KD or *ST18*-KD did not induce hormone co-expression of insulin, glucagon and somatostatin (Fig. 1i–t) or insulin, pancreatic polypeptide and somatostatin (ESM Fig. 2a–l). The levels of insulin or glucagon were not affected by KD (Fig. 1u, v), nor were beta cell TFs such as PDX1, MafA and NKX6.1 (ESM Fig. 2m–u).

### High levels of *MYT1* are required for beta cell survival under normal physiology

**Fig. 2:**
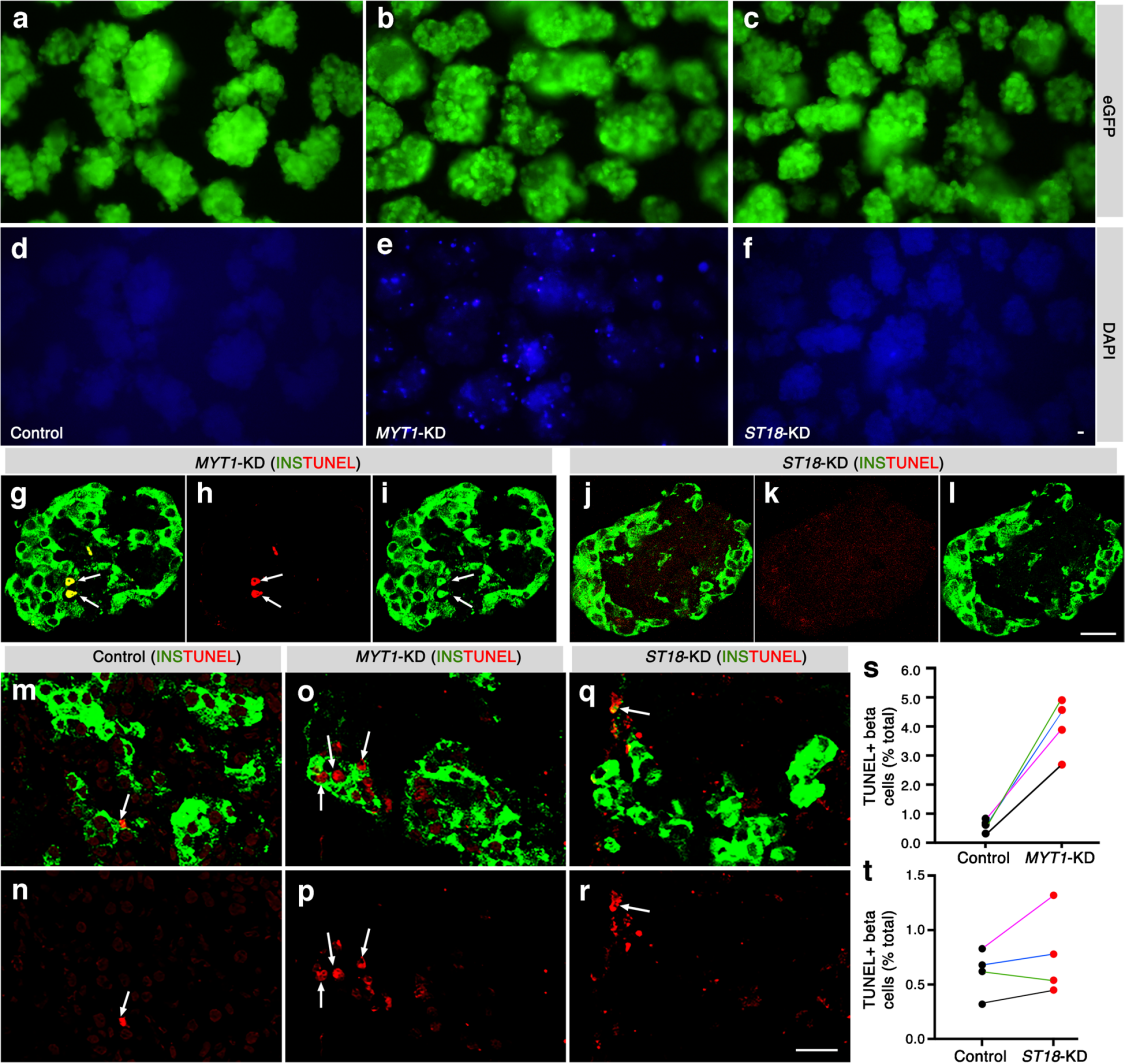
*MYT1*-KD, but not *ST18*-KD, causes the death of human beta cells in vitro and in vivo under normal physiological conditions. Freshly made PSIs, either control or with *MYT1*-KD or *ST18*-KD, were stained with DAPI to visualise dead cells. They were also examined for cell death with TUNEL assays before or 4 weeks after transplantation. (**a**–**f**) PSIs with control, *MYT1*-targeting or *ST18*-targeting shRNA expression (with green eGFP reporter) (**a**–**c**), with the respective corresponding DAPI staining (**d**–**f**). (**g**–**l**) TUNEL staining of *MYT1*-KD (**g**–**i**) and *ST18*-KD (**j**–**l**) PSIs. White arrows point at two dead cells with insulin-signals. (**m**–**r**) TUNEL assays and quantification of dead beta cells 4 weeks after PSIs were transplanted into the AEC of mice. Merged (**m**, **o**, **q**) and a single TUNEL channel (**n**, **p**, **r**) for each condition are shown. White arrows point at several dead insulin-positive cells. (**s**, **t**) The percentage of beta cells with TUNEL signals in control and KD PSIs with islets from four donors used. See ESM Table 9 for original cell numbers in each of the four donors. Each circle represents results from one donor, transplanted into 2–4 mice. The colour of the connecting lines indicate donor identity (black, blue, pink and green lines correspond to donor 1, 2, 4 and 8, respectively). Note the large effect size in (**s**), although no statistical significance was observed (*p*=0.06, analysed by Wilcoxon signed-rank test). Sum of positive, negative ranks: 10.00, 0.00. Connecting lines indicate islets from the same donor. Scale bars, 20 μm

Freshly prepared PSIs were stained with DAPI to label dead cells. PSIs with *MYT1*-KD but not *ST18*-KD contained DAPI^+^ cells (Fig. 2a–f). TUNEL assays detected DNA fragmentation in *MYT1*-KD beta cells, suggesting their apoptosis (Fig. 2g–l).

Because human beta cell death detected in vitro has sometimes not been shown in vivo [42], we examined how *MYT1*-KD or *ST18*-KD beta cells behaved as xenotransplants*.* PSIs were transplanted into the AECs of NSG-DTR mice. We found more (6.8 ± 0.83-fold) beta cell death upon *MYT1*-KD but not *ST18*-KD PSI transplants compared with control transplants 4 weeks after transplantation (Fig. 2m–t and ESM Fig. 3a–c) in all four donor samples tested (ESM Table 9), although the Wilcoxon signed-rank test did not produce a statistically significant *p* value (*p*=0.06).

### *ST18*-KD renders beta cells vulnerable to obesity-induced death

**Fig. 3:**
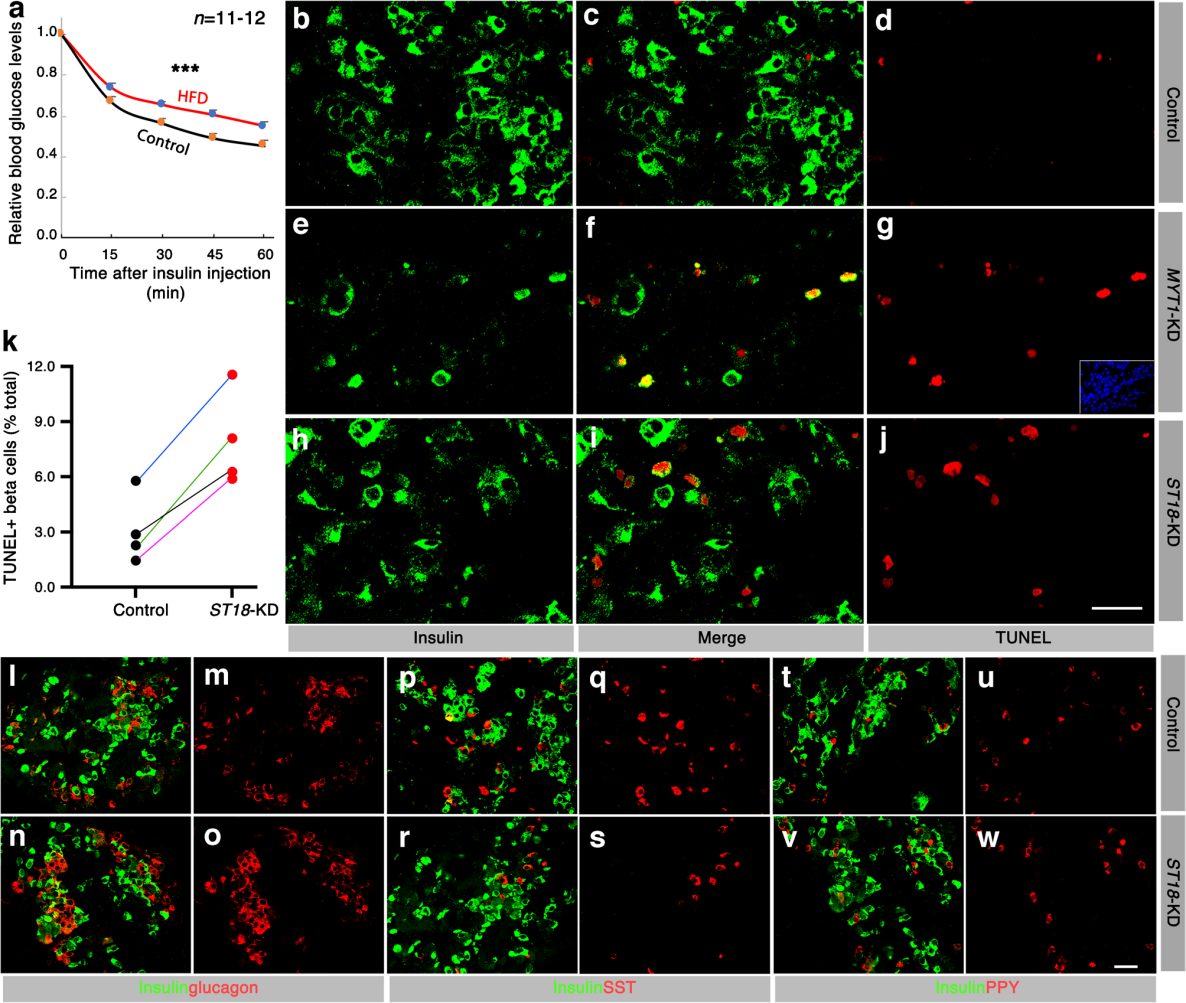
*ST18*-KD renders human beta cells vulnerable to HFD-induced death. Four weeks after the operation, mice transplanted with manipulated PSIs were fed with HFD for 12 more weeks. PSIs were then recovered for characterisation. (**a**) HFD-induced insulin resistance in recipient mice. The starting blood glucose of each mouse was normalised to ‘1’. The *p* value is from a linear mixed-effects model. ***p<0.001. (**b**–**k**) TUNEL assays of recovered transplants. Single insulin (**b**, **e**, **h**) and TUNEL (**d**, **g**, **j**), and merged panels (**c**, **f**, **i**), are shown. Inset in (**g**) shows DAPI staining in processed sections to visualise all nuclei. In (**k**), each circle represents data from one donor, including at least three transplanted mice. Connecting lines indicate islets from the same donor. The colour of the connecting lines indicated donor identity (black, pink, blue and green lines correspond to donor 1, 2, 4 and 8, respectively). See ESM Table 9 for the number of TUNEL^+^ or total beta cells counted in each donor sample, consistently showing higher proportions of TUNEL^+^ cells with *ST18-*KD. In (**k**), *p*=0.06, analysed by Wilcoxon signed-rank test is. Sum of positive, negative ranks are 10.00, 0.00. (**l**–**w**) Immunofluorescence images showing the co-expression of insulin and other hormones in recovered transplants, with merged images (**l**, **n**, **p**, **r**, **t**, **v**) and single glucagon (**m**, **o**), somatostatin (**q**, **s**) and pancreatic polypeptide (**u**, **w**) channels shown. Scale bars, 20 μm. PPY, pancreatic polypeptide; SST, somatostatin

We examined how *MYT1*-KD or *ST18*-KD affected human beta cell responses to obesity. Mice with PSI xenotransplants were fed with an HFD for 12 weeks, starting 4 weeks after transplantation. This induced insulin resistance but not overt diabetes (Fig. 3a). Consistent with the death of *MYT1*-KD beta cells without HFD treatment, very few insulin-positive cells were detected in any of the four batches of *MYT1*-KD transplants (Fig. 3b–g and ESM Fig. 3d–f). Unlike the lack of beta cell death in *ST18*-KD samples without HFD challenge (ESM Fig. 3g, h), HFD treatment induced beta cell death in all four batches of transplants by 2.73 ± 0.47-fold (Fig. 3h–k, ESM Fig. 3d–f and ESM Table 9), although the Wilcoxon signed-rank test did not reveal statistical significance (*p*=0.06). We did not detect insulin-positive cells activating other islet hormones (Fig. 3l–w).

### *MYT1*-KD or *ST18*-KD deregulates genes involved in cell death or secretion

**Fig. 4:**
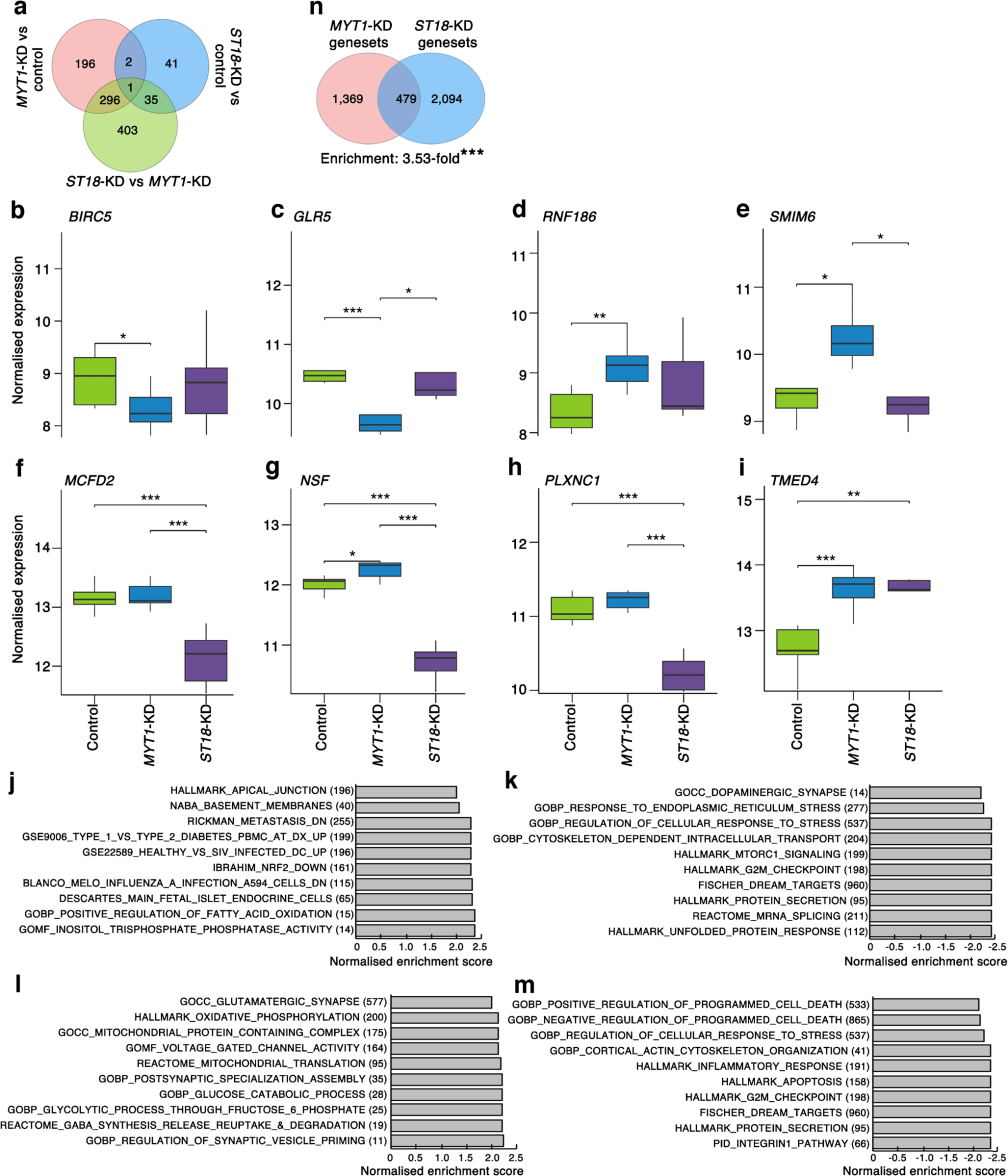
Human PSIs with *MYT1*-KD or *ST18*-KD deregulate overlapping but distinct genes/genesets, especially those involved in cell death and secretion. DESeq2 and GSEA were used to identify DEGs or dysregulated pathways. (**a**) A Venn diagram showing the number and overlapping status of DEGs when comparing the expression between samples of *MYT1-*KD vs controls, *ST18-*KD vs controls, and *ST18*-KD vs *MYT1*-KD. The single gene shared by all three DEG lists is *CEND1*, which is important for neuronal differentiation but its function in beta cells is unknown. (**b**–**i**) Expression of a few selected DEGs involved in cell death/apoptosis, stress response and secretion related genes in control, *MYT1*-KD and *ST18*-KD PSIs. The *y*-axis shows the normalised expression values after variance stabilising transformation (VST) in DESeq2. Results are shown (box and whisker plot) from four biological samples (two or three biological replicates per sample). The whiskers indicate the lower and upper limits of data. The boxes indicate the first and third quartiles, with the median indicated with a line within the box. *p<0.05, **p<0.01, ***p<0.001 (analysed by the Mann–Whitney *U* test [pairwise]). (**j**, **k**) Selected up- or downregulated pathways in *MYT1-*KD vs control PSIs*.* (**l**, **m**) Selected up- or downregulated pathways in *ST18-*KD vs control PSIs*.* For each geneset in (**j**–**m**), the numbers in parentheses indicate the number of leading-edge genes in that geneset. (**n**) Number of overlapping pathways between those affected by *MYT1*-KD or *ST18*-KD compared with controls (***p<0.001, hypergeometric analysis)

The above findings led us to hypothesise that MYT1 and ST18 use distinct genetic networks to regulate beta cell survival and/or function. We explored this using RNA-seq in control and KD PSIs. Out of the 34,266 genes expressed in PSIs (ESM Table 1), there were 495, 79 and 735 DEGs (>41.4% expression differences, statistically significant) between *MYT1-*KD vs control, *ST18-*KD vs control and *ST18-*KD vs *MYT1-*KD samples, respectively (Fig. 4a, ESM Fig. 4a–c and ESM Tables 10–12). The DEGs from *MYT1-*KD vs control PSIs were enriched for organelle membrane, granule, ion transport, etc., processes (ESM Table 13a). Microtubule binding, shown to regulate insulin secretion [43, 44], was enriched in the DEGs from *ST18-*KD vs control (ESM Table 13b). Similarly, the DEGs of *ST18-*KD vs *MYT1-*KD PSIs were enriched for regulation of membrane, secretion, death, etc. (ESM Table 13c).

We inspected the DEGs individually, focusing on those regulating cell death/apoptosis, stress response and secretion. Thirty-three of the 495 DEGs from *MYT1-*KD vs control have established roles in these processes (ESM Table 10). We found both upregulated and downregulated cell death/stress response genes, including both positive and native regulators. For example, *BIRC5* and *GLR5*, both of which inhibit cell death in response to increased cellular or excitatory stress (see ESM Table 10 for references), were downregulated in *MYT1-*KD PSIs (Fig. 4b, c). In contrast, *RNF186* and *SMIM6* were both upregulated, with RNF186 inducing cell death in response to ER stress and SMIM6 inhibiting it (Fig. 4d, e). From the 79 *ST18*-KD DEGs, nine were involved in cell death, stress response or secretion (ESM Table 11). For example, *MCFD2*, *NSF* and *PLXNC1*, all promoting secretion, were downregulated (Fig. 4f–h), while *TMED4*, inhibiting ER stress, was upregulated (Fig. 4i). These findings are consistent with the reduced cell viability or GSIS in *MYT1*-KD or *ST18*-KD PSIs, respectively.

### MYT1 and ST18 regulate common and unique gene sets in PSIs

GSEA revealed that *MYT1*-KD in PSIs resulted in statistically significant enrichment (>1.40-fold) across 1848 gene sets compared with controls (ESM Table 14). The upregulated gene sets regulated glucose metabolism, mitochondria, channels, γ-aminobutyric acid (GABA)-regulated secretion, etc. (Fig. 4j), which either promote GSIS (e.g. glucose metabolism [45]) or inhibit it (e.g. GABA synthesis [46]). The downregulated gene sets regulated stress response, apoptosis, etc. (Fig. 4k). Similarly, *ST18*-KD statistically significantly deregulated 2573 gene sets (i.e. pathways) over controls (ESM Table 15), including upregulated pathways in fatty acid oxidation, oxidative stress (NRF2-related), etc. (Fig. 4l), and downregulated pathways in ER stress, protein secretion, etc. (Fig. 4m). Consistent with the overlapping and different activities of the MYT TFs, *MYT1-*KD and *ST18-*KD shared 479 deregulated gene sets (ESM Table 15), representing a statistically significant 3.53-fold enrichment (*p*<0.001, Fig. 4n); there were also 1379 gene sets differentially expressed between the *MYT1-*KD and *ST18-*KD PSIs (ESM Table 16, ESM Fig. 4d, e). Also consistent with the roles of MYT TFs in stress response, both *MYT1*-KD or *ST18*-KD PSIs had deregulated ‘regulation_of_cellular_response_to_stress’ (Fig. 4k, m).

### scRNA-seq revealed ST18-dependent genes in beta cells

**Fig. 5:**
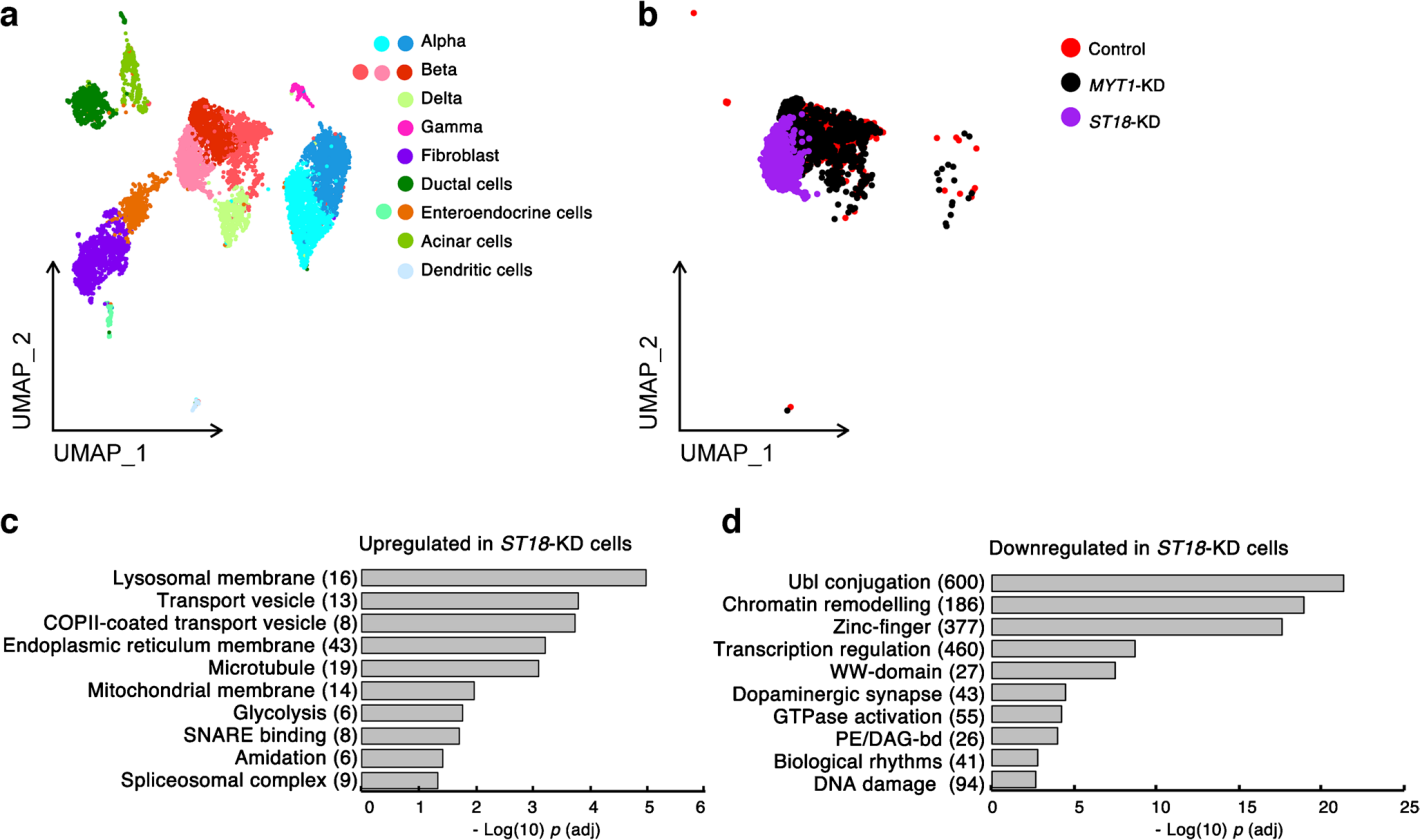
*ST18*-KD deregulates several processes involved in beta cell secretory functions. Freshly prepared PSIs were dissociated and used for InDrop scRNA-seq. (**a**, **b**) UMAPs showing the separation of cells in PSIs, including all cells (**a**) or beta cells only (**b**). (**c**, **d**) Pathways deregulated in *ST18*-KD human beta cells, composed of up- (**c**) or downregulated (**d**) genes in mutants. The number in parentheses following each process refers to the number of genes discovered. *p* values were adjusted with the Benjamini–Hochberg procedure

High-quality sequencing data, judged by the number of UMIs or numbers of genes identified per cell and the per cent of mitochondrial genes (ESM Fig. 5a), were obtained from one batch of PSIs, detecting all the major islet cell types and non-islet cells in PSIs (alpha, beta, delta and gamma) (Fig. 5a and ESM Fig. 5b, c). These contained a total of 536, 906 and 661 beta cells in control, *MYT1*-KD and *ST18*-KD samples, respectively. There was no clear separation between control and *MYT1*-KD beta cells (Fig. 5b); neither was there any difference in their *MYT1* expression, despite the presence of 687 DEGs between them (ESM Table 17). These findings suggest that the *MYT1*-KD scRNA-seq dataset was enriched for cells without *MYT1*-KD due to the selective apoptosis of *MYT1*-deficient beta cells, which could exert non-cell-autonomous effects on the surviving beta cell population [47].

Control and *ST18*-KD beta cells segregated into clearly distinct clusters and displayed 3284 DEGs (>20% difference), including *ST18* (Fig. 5b and ESM Table 18). *ST18*-KD upregulated several membrane-related processes, SNARE binding and mitochondrial membrane (Fig. 5c) while it downregulated Ubl conjugation, biological rhythms, etc. (Fig. 5d). Consistent with the death of *ST18-*KD beta cells in vivo under stress, *HSPA1A,* the overexpression of which led to mouse beta cell death in vivo [18], was activated in *ST18-*KD beta cells (ESM Table 18).

### *ST18*-KD compromises glucose-stimulated Ca^2+^ influx in beta cells

To test whether the increase in mitochondrial membrane proteins in *ST18-*KD beta cells results in higher mitochondrial activities, we used super-resolution microscopy to compare mitochondrial volume in control and *ST18*-KD beta cells (ESM Methods: Mitochondria assays). The *ST18*-KD beta cells had higher mitochondrial volume (18.1 ± 2.6%) in all four batches of donor islets tested (ESM Fig. 6a–e), although this did not result in higher mitochondrial transmembrane potential in mutant cells (ESM Fig. 6f). We also used super-resolution microscopy to compare the numbers of insulin granules and found no difference between control and *ST18*-KD beta cells (ESM Fig. 6g–k).

**Fig. 6:**
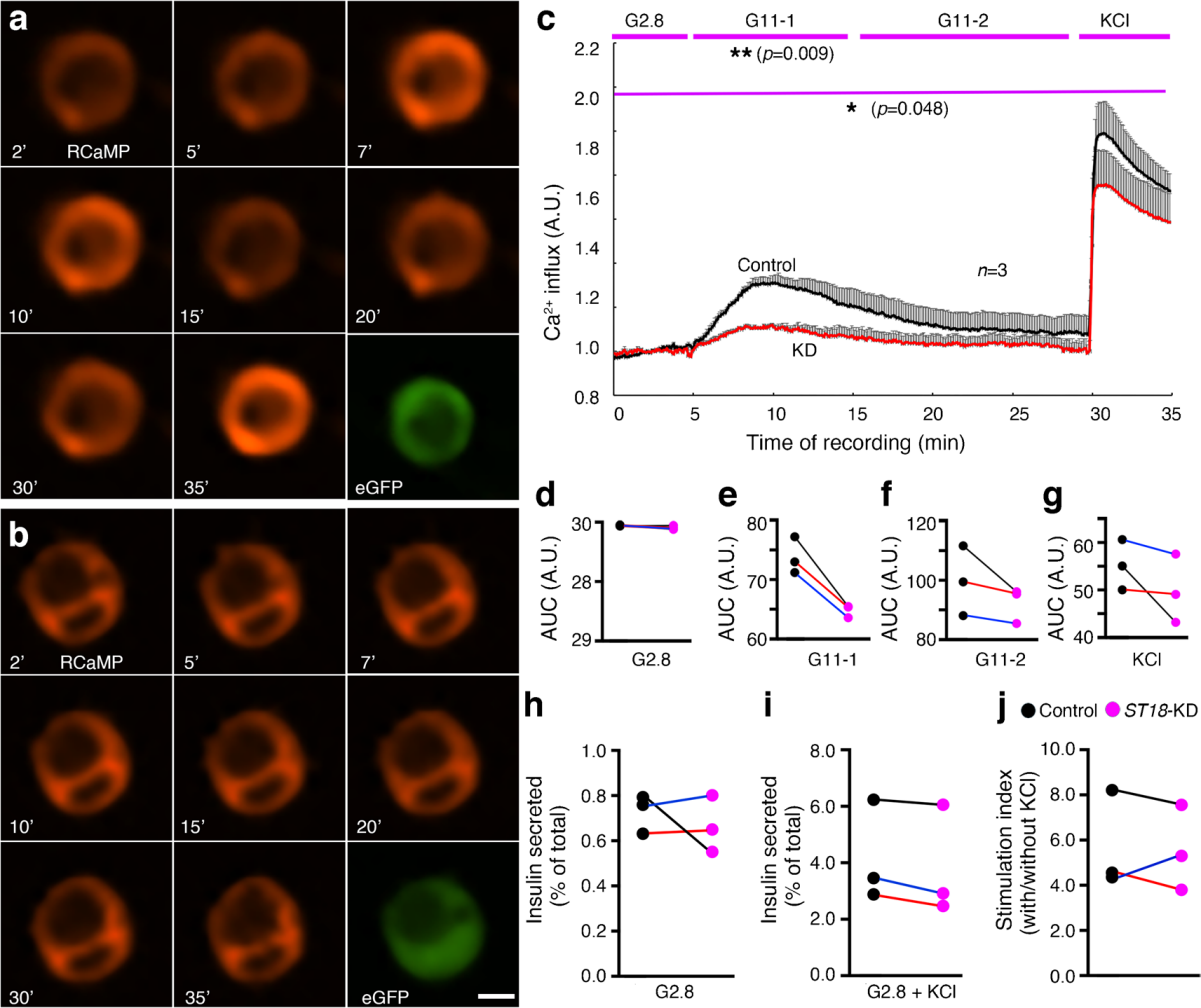
*ST18*-KD compromises glucose-induced Ca^2+^ influx in beta cells. (**a**–**g**) The effect of *ST18*-KD on Ca^2+^ influx in beta cells, shown by the intensity change of RCaMP fluorescence over time after addition of 11 mmol/l glucose or 30 mmol/l KCl (at 5 min or 30 min, respectively). One control (**a**) and one *ST18-*KD cell (**b**) are shown. eGFP expression (green) was used to identify cells with control or targeting shRNA expression. Scale bar, 5 μm. (**c**–**g**) RCaMP fluorescence intensity changes over time in beta cells of three donors; 126, 13 and 127 control cells (black; from donor 5, 6 and 7, respectively) and 138, 102 and 123 *ST18-*KD cells (red/pink; from donor 5, 6 and 7, respectively) were assayed. The results from each donor’s cells were averaged and treated as one sample (represented by one circle in **d**–**g**). The temporal mean + SEM values are presented in (**c**). *p* values were from a linear mixed-effects model, analysed at separate phases of the recording (G2.8, 10 min of G11 [G11–1], 15 more min of G11 [G11–2], and 5 more min of 30 mmol/l KCl) or the entire process (**p=0.009; *p=0.048). The relative AUC for each phase is presented (**d**–**g**). (**h**, **i**) Insulin secretion in control and *ST18*-*KD* PSIs in response to KCl-induced depolarisation in 45 min windows. Each circle represents the average of three or four technical replicates from one donor. The connected circles indicate results from the same donor. (**j**) A representation of the combined data in (**h**) and (**i**)*,* showing the stimulation index of PSIs in response to KCl. The colour of the connecting lines in (**d**–**j**) indicate the donor identity (black, donor 5; red, donor 6; blue, donor 7). G11, 11 mmol/l glucose

A downregulated process in the *ST18*-KD beta cells was ‘dopaminergic synapse’ (Fig. 5d), including voltage-gated Ca^2+^ channel genes *CACNA1A*, *CACNA1B*, *CACNA1C* and *CACNA1D* (ESM Table 18). In addition, several K^+^ and Na^+^ channel genes were affected by *ST18*-KD (ESM Table 18). Consequently, the *ST18*-KD compromised glucose-induced Ca^2+^ influx, with the most significant effect occurring within the first 10 min of glucose stimulation (by 12.1 ± 1.6% of total cytoplasmic Ca^2+^) (Fig. 6a–c). The AUC of the Ca^2+^ influx in these processes did not reveal statistically significant changes, likely because the glucose-induced Ca^2+^ influx accounts for only a small portion of the total Ca^2+^ (Fig. 6d–f). *ST18-*KD beta cells showed statistically insignificant changes in KCl-induced Ca^2+^ influx (Fig. 6c, g) and insulin secretion (Fig. 6h, i). These results are consistent with the conclusion that the defective glucose-induced Ca^2+^ influx, a major cause of the reduced GSIS in *ST18-*KD beta cells, is likely a combined result of deregulated Ca^2+^, K^+^ and Na^+^ channels, and other processes. Note that *ST18-*KD beta cells upregulated several SNARE-binding-protein genes that usually promote GSIS (*NSF*, *SYT5*, *SYT7*, *STX1A*, *STXBP1* and *VAMP2*) (ESM Table 18). These upregulated genes may be unable to compensate for reduced Ca^2+^ influx to maintain GSIS, or they may not be the limiting factors for secretion.

### Human MYT1- and ST18-regulated genes are statistically enriched for type 2 diabetes-associated genes

We tested whether MYT1 or ST18 regulated type 2 diabetes risk genes. We used several published data sets: (1) the 3319 type 2 diabetes risk genes in GWAS Catalog [41], including 2047 PSI-expressed genes and 1806 beta cell-expressed genes (ESM Tables 3, 4); (2) the 849 type-2-diabetes-associated genes from Suzuki et al [1], including 665 PSI-expressed genes (ESM Table 5) and 621 beta cell-expressed genes (ESM Table 6); and (3) the 709 islet DEGs (686 expressed in PSIs, ESM Table 7) or 365 beta cell DEGs (ESM Table 8) between islets from control donors and donors with type 2 diabetes.

**Fig. 7:**
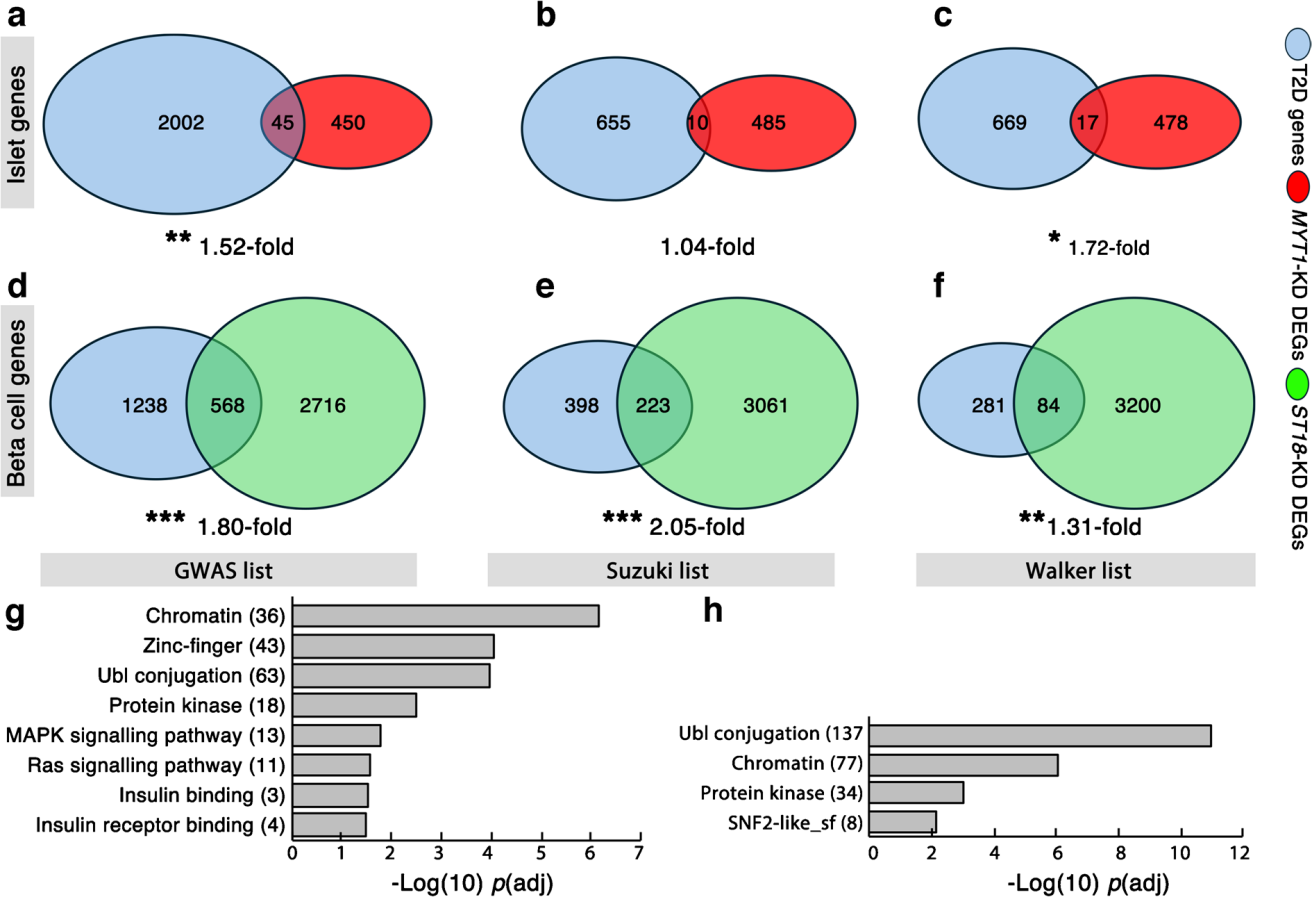
DEGs induced by *MYT1*- or *ST18*-KD are enriched for type-2-diabetes-associated genes. (**a**–**c**) Venn diagrams showing the number of overlapping genes between the type-2-diabetes-associated genes and the DEGs induced by *MYT1*-KD in PSIs. All genes with detectable expression in PSIs (ESM Table 1) were used as the starting population in overlapping studies. The enrichment fold and *p* values were from hypergeometric tests. The GWAS gene list (ESM Table 3), the Suzuki gene list (ESM Table 5) and the Walker gene list (ESM Table 7) were used separately. (**d**–**f**) As for (**a**–**c**), with the DEGs from *ST18*-KD vs control beta cells and the type-2-diabetes-associated genes only detected in beta cells from the three separate studies (ESM Tables 4, 6 and 8). *p<0.05, **p<0.01, ***p<0.001 (all from hypergeometric analysis). (**g**) Biological processes enriched in the shared genes between type-2-diabetes-associated beta cell genes (GWAS list) and *ST18*-KD-induced DEGs in beta cells from scRNA-seq data. (**h**) As for (**g**), except that the type-2-diabetes-associated genes were from the Suzuki studies. In (**g**, **h**), the *p* values were adjusted with the Benjamini–Hochberg procedure and the numbers in the parentheses following each pathway indicate the number of genes discovered in that pathway. T2D, type 2 diabetes

Among the 495 DEGs between control and *MYT1*-KD PSIs, we found 45, 10 and 17 genes that overlapped between these DEGs and the GWAS, Suzuki and Walker PSI lists. This represented a respective 1.52-fold (*p*=0.011), 1.04-fold (statistically non-significant) and 1.72-fold (*p*=0.023) enrichment over random gene distribution (hypergeometric tests) (Fig. 7a–c). For ST18-regulated genes, we used beta cell-specific DEGs, which shared 568, 223 and 84 genes with the beta cell-expression GWAS, Suzuki and Walker lists, all representing statistically significant enrichments (≥1.31-fold enrichment, *p*≤0.004, hypergeometric tests) (Fig. 7d–f).

Last, we examined the pathways shared by type 2 diabetes risk and ST18-regulated beta cell genes. Among those shared by the GWAS list and *ST18-*KD DEGs, chromatin, zinc fingers, Ubl conjugation, several kinase signalling processes, and insulin-binding processes were statistically enriched (*p*_adj_<0.05) (Fig. 7g and ESM Table 18). Among those shared by the Suzuki list and the *ST18*-KD DEGs, Ubl conjugation, chromatin, kinase and SNF2-like processes were statistically enriched (Fig. 7h).

## Discussion

In this study, we showed that *MYT1*-KD induced human beta cell apoptosis, whereas *ST18*-KD compromised GSIS under normal physiological conditions. *ST18*-KD also promoted obesity-induced beta cell death. At the molecular level, MYT1 and ST18 regulated common and unique genes that were enriched for those associated with type 2 diabetes risk.

Our overall findings suggest that the MYT TFs integrate genetic and nutritional factors to prevent beta cell failure and type 2 diabetes. In beta cells, obesity-associated high glucose or NEFA lead to overproduction of unfolded proteins or reactive oxygen species, which are cleared via stress response [14, 48]. Yet, overactivation of this response also leads to beta cell failure. In mice, the MYT TFs prevent this latter scenario [18]. We now show that these MYT TF functions can be extended to human beta cells, likely involving a few type-2-diabetes-associated genes and other genes that regulate beta cell death and/or function [49]. These new results, together with the regulation of MYT1 and ST18 by nutrients in human beta cells [18], the association of the MYT genes with type 2 diabetes [41], and the reduced expression of MYT TFs in type 2 diabetes beta cells [18], suggest MYT TFs as key tunable repressors of obesity-induced beta cell failure. Manipulating MYT1 and ST18 activities may be explored to prevent this failure and the development of type 2 diabetes.

Our studies revealed the different and complementary functions of MYT1 and ST18. MYT1 is essential for beta cell survival whereas ST18 is essential for GSIS under normal physiological conditions. Yet ST18 also has pro-survival activity under metabolic stress. The unique and overlapping pathways regulated by these factors corroborate these observations. These combined findings highlight a mechanism whereby multiple processes could regulate the same cellular function under different physiological conditions, so that different phenotypes would prevail under different conditions when a specific process was perturbed.

Our findings also highlighted the species-specific roles of the MYT TFs, despite their high levels of sequence conservation [18]. *MYT1-*KD caused human beta cell death, while *Myt1* inactivation compromised mouse beta cell function but not survival [21]. Similarly, *ST18*-KD results in human beta cell death under obesity, yet a mouse ST18 protein level reduction did not [50]. Furthermore, mouse beta cells with *Myt1* single mutation and MYT TF triple gene mutations had compromised identity under metabolic stress but human beta cells with *MYT1*-KD or *ST18*-KD did not. These findings highlight the species-specific functions of MYT TFs in maintaining beta cell identity.

Several issues remain to be addressed. First, gene KD may not fully reveal the molecular changes underlying beta cell dysfunction or apoptosis due to donor differences, varying gene KD levels with shRNA, and the sample size (*n*=4) being limited by donor islet availability. Thus, the identified DEGs and differentially expressed gene sets are considered preliminary, and correlating gene expression changes with cellular defects requires caution. Second, how MYT1 regulates beta cell viability remains incompletely understood. We detected statistically significant MYT1 reduction and altered expression of several cell-death regulators in *MYT1-*KD PSIs, likely due to the inclusion of dying cells in these samples. Yet, we could not detect *MYT1* downregulation in beta cells using scRNA-seq, likely because dying cells were excluded. Future studies of the death-regulating genes in beta cells are needed. Third, how *ST18* regulates Ca^2+^ influx is unclear. *ST18-*KD deregulated the expression of several Ca^2+^, K^+^, and Na^+^ channel-coding genes, likely contributing to the defective glucose-induced Ca^2+^ influx. Yet KCl-induced depolarisation did not statistically significantly alter Ca^2+^ influx, suggesting that ion channels (especially Ca^2+^ channels) alone cannot explain the defective Ca^2+^ influx. Other Ca^2+^-regulating processes controlled by ST18 need to be explored in the future. Lastly, the direct transcriptional targets of MYT1 and ST18 are unknown; this needs to be addressed in the future using chromatin-binding-based studies.

## Supplementary Information

Below is the link to the electronic supplementary material.

ESM (PDF 16320 KB)

ESM Tables (XLSX 3545 KB)

## Data Availability

The RNA-seq data were deposited in the Gene Expression Omnibus (GEO) (GSE307815 for scRNA-seq and GSE307751 for bulk RNA-seq). They are now freely available from the following sites: www.ncbi.nlm.nih.gov/geo/query/acc.cgi?acc=GSE307815; and www.ncbi.nlm.nih.gov/geo/query/acc.cgi?acc=GSE307751. GG is the guarantor of the data, and he will fulfil reasonable resource and reagent requests.

## Abbreviations

AEC: Anterior eye chamber
DEG: Differentially expressed gene
ER: Endoplasmic reticulum
G1: 1 mmol/l glucose
G16.7: 16.7 mmol/l glucose
G2.8: 2.8 mmol/l glucose
GABA: γ-Aminobutyric acid
GO: Gene Ontology
GSEA: Gene set enrichment analysis
GSIS: Glucose-stimulated insulin secretion
HFD: High-fat diet
KD: Knockdown
MYT TF: Myelin transcription factor
PSI: Pseudo-islet
scRNA-seq: Single-cell RNA-seq
