# Supplementary material for: Diabetes-associated MYT1 and ST18 genes regulate human beta cell insulin secretion and survival via other diabetes risk genes
Source: Diabetologia. 2026 Jun 5;69(9):2554–68. doi: 10.1007/s00125-026-06757-8 (PMC13424723; doi:10.1007/s00125-026-06757-8)
Supplement: Supplementary file 1 — ESM (PDF 16320 KB) [file 125_2026_6757_MOESM1_ESM.pdf]

Summary of Electronic Supplementary Materials (ESMs):

1) Methods.
2) ESM table description (one file with 18 Tabs).
3) ESM Figures and legends (six figures).
4) Human-Islet-Checklist.

Methods:

HEK293 cell culture-based reporter assays

The HEK293 cell line, cultured in DMEM supplemented with 10% fetal bovine serum, was used to select shRNAs that effectively target *MYT1* or *ST18,* respectively. Briefly, four candidate shRNAs were designed for each gene, each expressed together with mCherry in one tester plasmid (ESM Fig. 1a). Reporters expressing an eGFP cDNA fused with a shRNA target-sequence were also made (ESM Fig. 1a). Each set of the tester and reporter plasmids was then co-transfected into HEK293 cells using Lipofectamine (MIRUS). The eGFP-to-mCherry protein ratios and eGFP mRNA levels were compared to identify the most effective for *MYT1*-KD and *ST18*-KD, respectively (ESM Fig. 1b-q). Note that the eGFP-to-mCherry protein ratio was assayed by fluorescence image capture and quantification with Image J (NIH, USA), while the eGFP mRNA ratio was assayed using Real-time quantitative RT-PCR (see methods in the main text). Also, for eGFP mRNA assays, we further normalized the eGFP mRNA levels against the copy number of reporter plasmids. Oligos used were: for plasmid DNA copy number: 5’-tccttctagtgtagccgtagtt-3’+ 5’-cagccactggtaacaggattag-3’; for eGFP mRNA: 5’-gaaccgcatcgagctgaa-3’+ 5’-tgcttgtcggccatgatatag-3.

scRNA-seq quality control

Dissociated PSIs with ~80-90% single cells and <5% dead cells were used for InDrop, sequenced with Novaseq 6000 (Illumina) with ~120 million reads per sample. DropEst was used to generate count matrices. Cells with low unique mapping reads (<500), low proportion of expressed genes (<100), or high proportion of mitochondrial RNAs (>10%) were excluded (4).

Mitochondria assays

Human islet cells were dissociated into single-cell suspensions and attached to glass-bottom plates in the presence of eGFP-expressing lentivirus that also expressed control or targeting shRNAs. Four-five days later, cells were fixed and stained with antibodies against insulin and Tomm20. Super-resolution images were acquired with Leica-880 LSM or Nikon-NSPARC optics (z-stacks, ~0.2 μm per slice) and quantified using ImageJ (NIH, USA). To examine mitochondrial transmembrane potential, PSIs with KD were dissociated into single cells and incubated with MitoView-633 for ~15 minutes under 20mM glucose. Flow cytometry was then performed to assay the MItoView 633 fluorescence levels, which positively correlate to mitochondrial transmembrane potential (PMC10627182/PMID: 37936577).

Supplementary Table. Spreadsheet for gene expression analysis and TUNEL-positive beta cell counting.

ESM Table 1: Complete list of genes with detectable expression in human PSIs.

ESM Table 2: Complete list of genes with detectable expression in human beta cells.

ESM Table 3: PSI-expressed T2D-associated genes (GWAS catalog).

ESM Table 4: Beta-cell-expressed T2D-associated genes (GWAS catalog).

ESM Table 5: PSI-expressed T2D-associated genes (Suzuki list).

ESM Table 6: Beta-cell-expressed T2D-associated genes (Suzuki list).

ESM Table 7: PSI-expressed T2D-altered genes (Walker list).

ESM Table 8: Beta-cell-expressed T2D-altered genes (Walker list).

ESM Table 9: Numbers of TUNEL+ beta and total beta cells in recovered PSI xenotranslpants after MYT1- or ST18-KD and transplantation into mice. The data for four batches of donor islets were presented separately.

ESM Table 10: DEGs between control and *MYT1*-KD PSIs.

ESM Table 11: DEGs between control and *ST18*-KD PSIs.

ESM Table 12: DEGs between *ST18*-KD and *MYT1*-KD PSIs.

ESM Table 13: Pathways/processes deregulated in pairwise comparisons between *MYT1*-KD, *ST18*-KD, and controls from Deseq2.

ESM Table 14: Deregulated genesets in *MYT1*-KD vs control PSIs.

ESM Table 15: Deregulated genesets in *ST18*-KD vs control PSIs.

ESM Table 16: Deregulated genesets in *ST18*-KD vs *MYT1*-KD PSIs.

ESM Table 17: DEGs between control and *MYT1*-KD single beta cells.

ESM Table 18: DEGs between control and *ST18*-KD single beta cells.

Supplementary Figures:

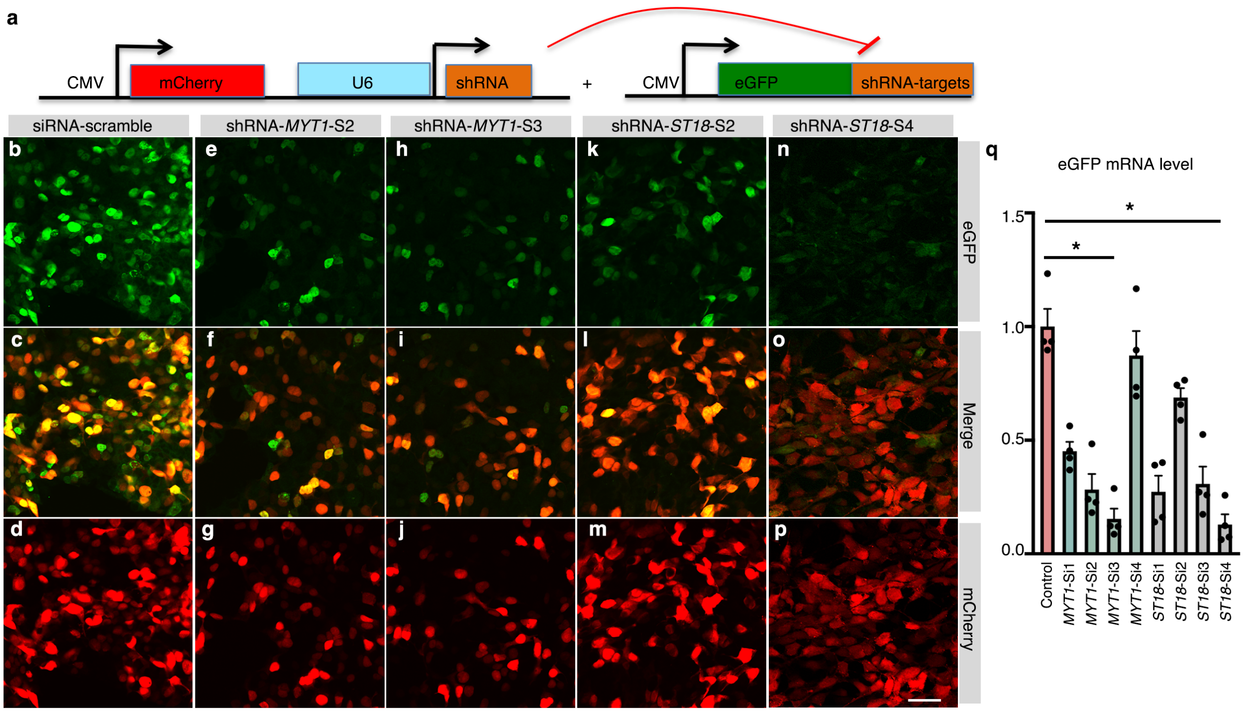

ESM Fig. 1. Identification of shRNAs for human *MYT1* and *ST18* KD. Four shRNA sequences were tested for each gene. (**a**) A diagram showing the tester (expressing shRNA and mCherry) and reporter (expressing eGFP cDNA and shRNA targeting a site) constructs. (**b**-**p**) Reporter assays showing the different efficacy of shRNA KD of reporter eGFP. Note that images of two MYT1 shRNA results (MYT1-Si2 and MYT1-Si3) and two ST18 shRNA results (ST18-Si2 and ST18-Si4) were included. (**q**) RT-PCR results to examine the mRNA downregulation by all eight targeting shRNAs. The control was a CMV-driven scrambled RNA. *: $p<0.05$, Mann-Whitney U test (sum of ranks: 26, 10). Bar, 20 µm.

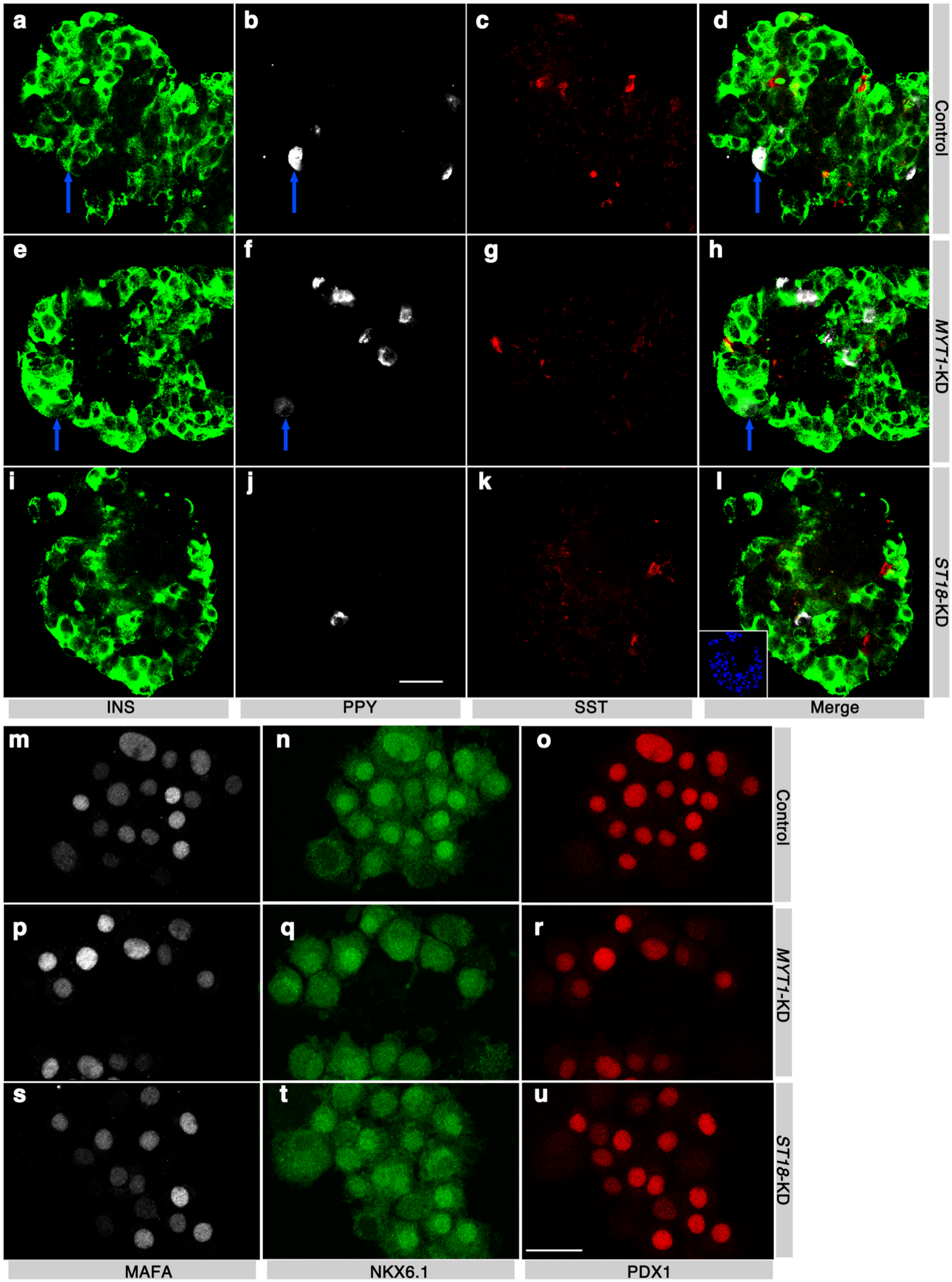

ESM Fig. 2. *MYT1*- or *ST18*-KD does not induce co-expression of islet hormones or alter the expression of several TFs in β cells. (**a–l**) Insulin co-expression with pancreatic polypeptide (PPY) and somatostatin (SST) in freshly prepared PSIs. Inset in l is a DAPI staining to locate all nuclei. (**m–u**) MAFA, NKX6.1, and PDX1 expression in control and KD-ed cells. (**a–i**) Single optical slices were shown. (**m–u**) Z-projections of the entire cell were shown. Blue arrows in **a–d** or **e–h** point at two cells co-expressing INS and PPY. Bars, 20 μm.

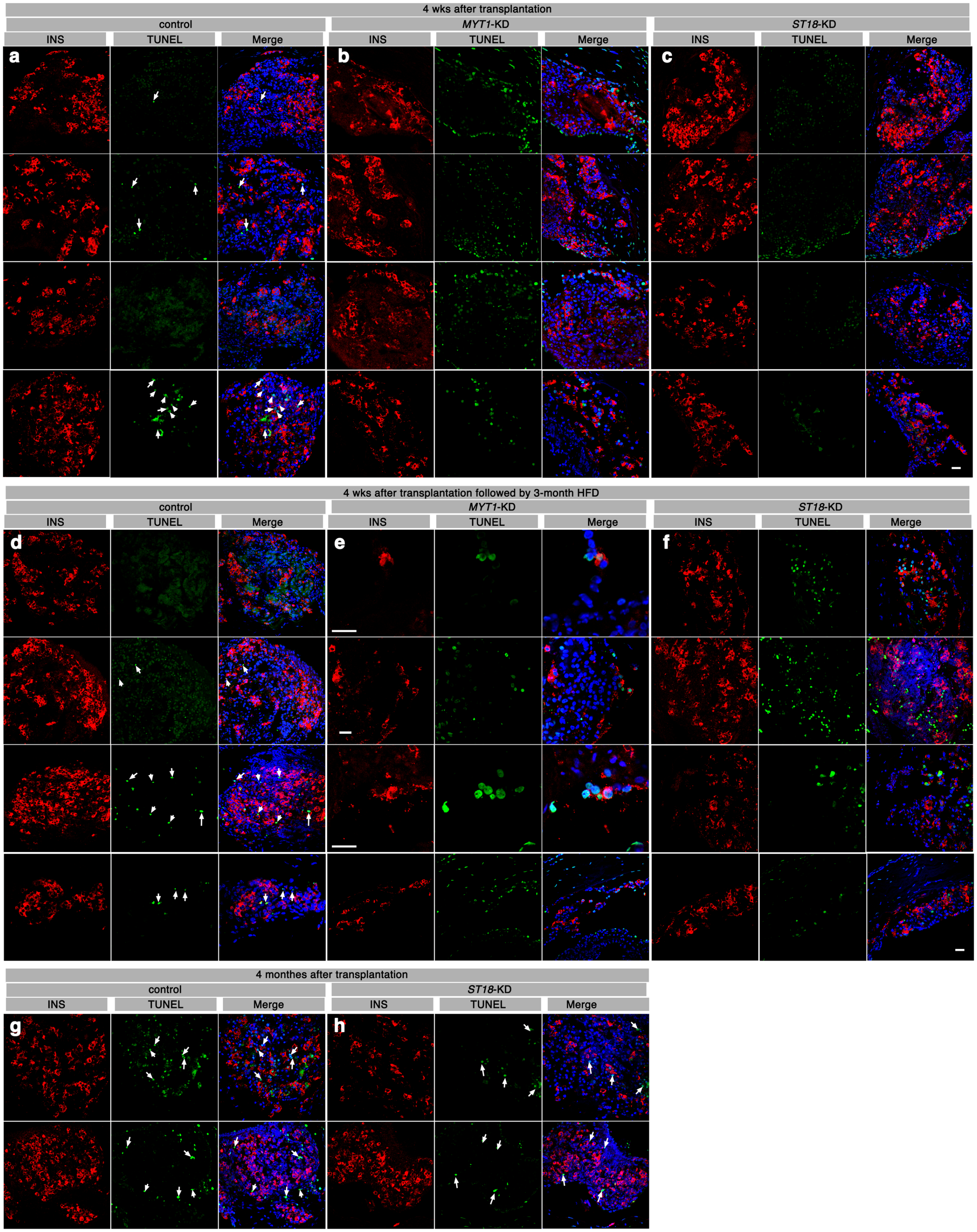

ESM Fig. 3. β-cell death with *MYT1*- or *ST18*-KD. (**a–c**) TUNEL staining 4 weeks after PSI transplantation. Each row used PSIs from one donor. Insulin, TUNEL, and merged images (with DAPI) were included. (**d–f**) and (**g, h**), PSIs after 3 additional months of HFD (**d–f**) or control diet feeding (**g, h**). White arrows in **a, d, g,** and **h,** examples of TUNEL$^+$insulin$^-$ cells. Bars, 20 μm, all panels shared the same scales (except the few in **e**, labeled separately).

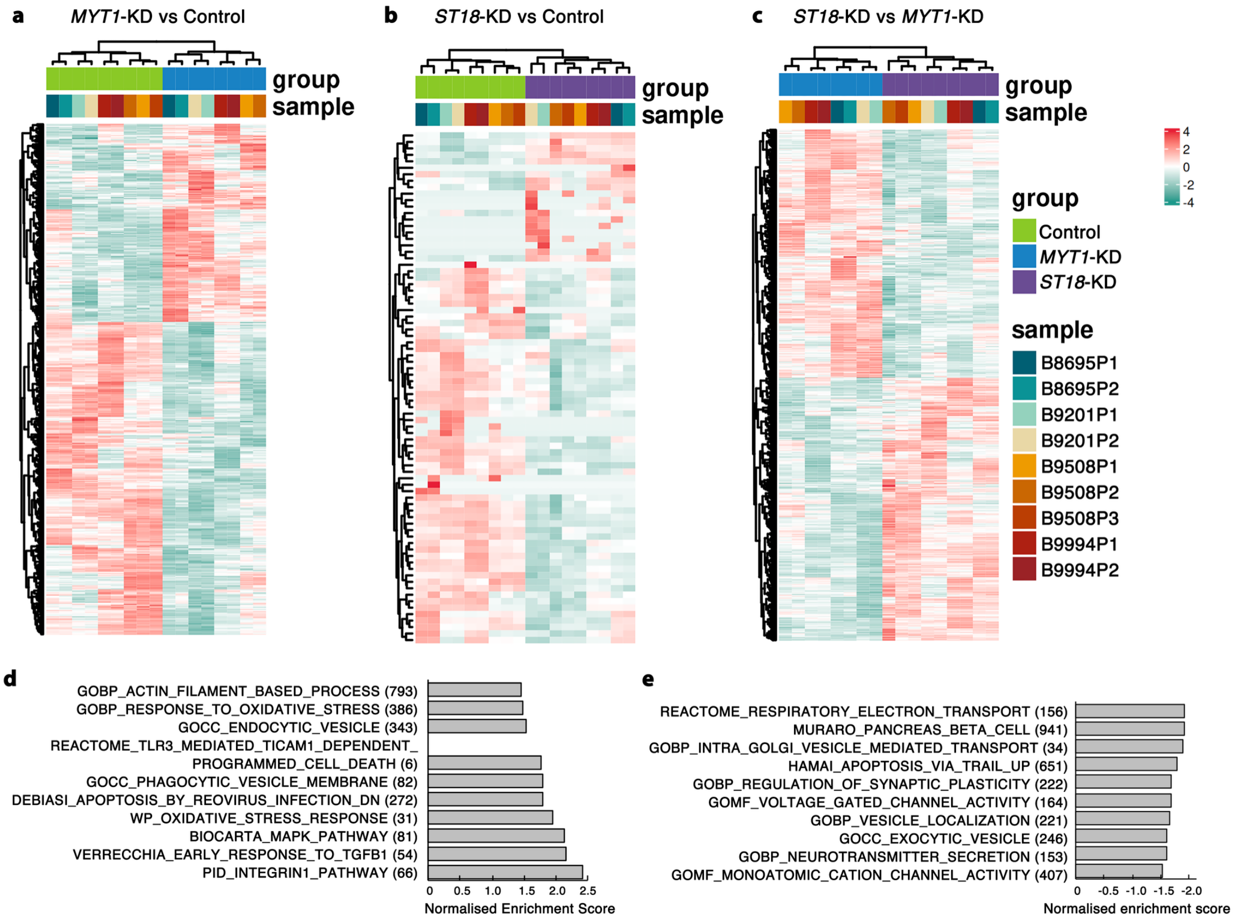

ESM Fig. 4. DEGs and changed genesets in pairwise comparison between control, *MYT1*-KD, and *ST18*-KD PSIs. (**a**–**c**) Hierarchical clustering of DEGs (from Deseq2) between control–*MYT1*-KD (**a**), control–*ST18*-KD (**b**), and *MYT1*-KD–*ST18*-KD samples (**c**). Technical replicates for each donor were included (the same designation corresponds to those deposited RNA-seq raw data). (**d, e**) Up- or down-regulated gene sets in *ST18*-KD over *MYT1*-KD PSI samples. Ten examples of each category were shown (see ESM Table 15 for the complete list).

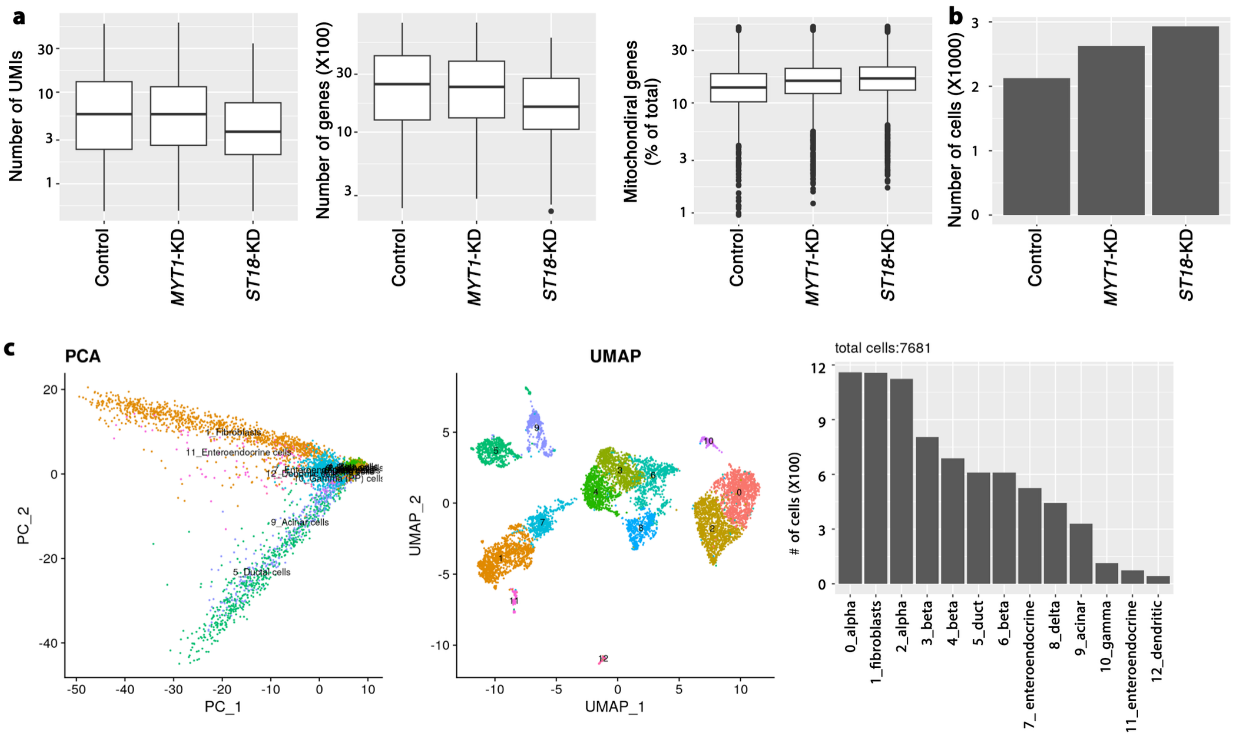

ESM Fig. 5. Quality controls showing the sc-RNAseq for one batch of human PSI (control, *MYT1*-KD, or *ST18*-KD). (**a**) The number of UMIs or genes per cell and the % of mitochondrial specific genes (higher % of mitochondrial genes indicates unsuccessful scRNA-seq). (**b**) The total number of cells sequenced in three samples. (**c**) Principal-component plots, or UMAPs, and bar graphs showing the distribution and numbers of different islet cell types. Note that we identified two alpha-cell subtypes and three beta-cell subtypes. These subtypes were not pursued in this study.

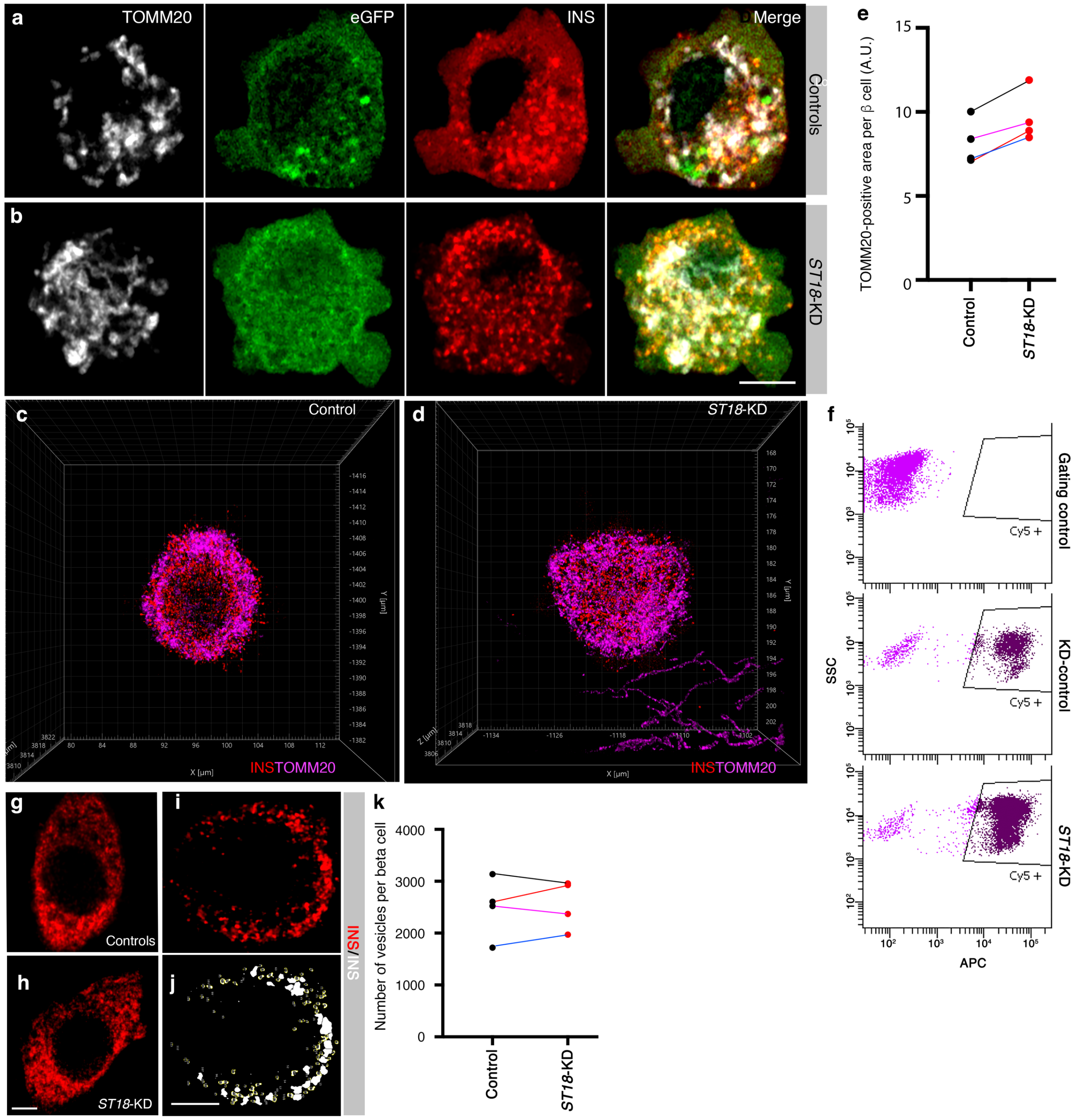

ESM Fig. 6. *ST18*-KD increased mitochondrial volume but did not affect mitochondrial transmembrane potential and insulin granule production. (**a–e**) Mitochondrial volume in each β cell after *ST18*-KD. (**a, b**) Single slice of confocal images (white: Tomm20, mitochondrial membrane; green, shRNA expression; red, insulin). (**c, d**) 3-D projections (IMARIS) of insulin granules and mitochondria in control and *ST18*-KD beta cells. (**e**) Quantification of mitochondrial volume in each beta cell, represented by the total mitochondrial area on all optimal slices. Results from four donors were presented, with those from the same donor connected by lines of different colors (black: donor #5; red: donor #6; blue: donor #7; pink: donor #8, see the Human-Islet-Checklist below). Ten to 14 beta cells were counted for each sample (donor/genotype). In **e***,* beta cells with *ST18-KD* showed higher mitochondrial volume in all four donor islet batches [*p*=0.06 with four donor data sets combined, a Wilcoxon signed rank test (sum of positive, negative ranks: 10, 0]. (**f**) A typical flow cytometry assay of transmembrane potential in mitochondria of PSI cells (indicated by the Cy5 intensity). Note the unequal distribution of gated cells, likely reflecting the different islet

cell types in PSI. (**g–k**) Quantification of the number of vesicles in each beta cell. (**g, h**) Typical insulin vesicles in control and *ST18*-KD beta cells. (**I, j**) Quantification method, with the non-contacting vesicles counted by ImageJ and contacting vesicles counted manually (**j**). Beta cells from four donors were counted. For each donor, 10-17 beta cells were examined. All optical slices that were 0.88 micrometers apart were counted to avoid double-counting vesicles. In **k**, the combined results of four donors were presented, with those of the same donor connected by lines of different colors (black: donor #5, red: donor #6, blue: donor #7, pink: #8). Bar, 5 μm.

Human-Islet-Checklist

Checklist for reporting human islet preparations used in research
Adapted from Hart NJ, Powers AC (2018) Progress, challenges, and suggestions for using human islets to understand islet biology and human diabetes. Diabetologia https://doi.org/10.1007/s00125-018-4772-2

**Islet preparation**	**1**	**2**	**3**	**4**	**5**	**6**	**7**	**8**
Unique identifier	**RP-003**	**SAMN31645455**	**SAMN37350251**	**SAMN28157682**	**SAMN34130383**	SAMN49948188	SAMN50225209	**SAMN36704819**
Donor age (years)	48	68	43	37	42	54	53	52
Donor sex (M/F)	F	M	F	M	F	M	F	M
Donor BMI (kg/m^2)	22.3	30.7	29.9	30.3	29.3	31.6	33.4	25.9
Donor HbA_{1c} or other measure of blood glucose control	5.0	5.4	5.2	5.0	5.2	5.8	5.8	4.9
Origin/source of islets[b]	NORTON ISLET CENTER	IIDP	IIDP	IIDP	IIDP	IIDP	IIDP	IIDP
Islet isolation centre	NORTON ISLET CENTER	Prado	Wisconsin	SC-ICRC	Prado	Imagine islet Center	SC-ICRC	Imagine
Donor history of diabetes? Please select yes/no from drop down list	No	No	No	No	No	No	No	No
Diabetes duration (years)	NA	NA	NA	NA	NA	NA	NA	NA
Glucose-lowering therapy at time of death[c]	NA	NA	NA	NA	NA	NA	NA	NA

Donor cause of death	Head Trauma	Anoxia	Head trauma	stroke	*Cerebrovascular/stroke*	*Cerebrovascular/stroke*	*Cerebrovascular/stroke*	stroke
Warm ischaemia time (h)	9 minutes	None	None	None	None	None	21 minutes	None
Cold ischaemia time (h)	9 hours 8 mins	10 hours 20 minutes	5 hours	7 hours 20 minutes	none	2 hour 50 minutes	9 hour 53 minutes	6 hours 10 minutes
Estimated purity (%)	90	85	90	80	90	85	95	80
Estimated viability (%)	95	90	95	96	90	90	95	95
Total culture time (h)[d]	60 hours	72 hours	92 hours	96 hours	118 hours	64 hours	46 hours	70 hours
Glucose-stimulated insulin secretion or other functional measurement[e]	GSIS, Stimulation index (SI) is 2.1	GSIS, SI is 3.5	GSIS, SI 3.4	GSIS, SI is 2.3	GSIS, SI, 3.2	GSIS, SI: 2.6	GSIS, SI: 3.1	GSIS, SI: 2.8
Handpicked to purity? Please select yes/no from drop down list	Yes	Yes	Yes	Yes	Yes	Yes	Yes	Yes
Additional notes	For Ca2+ recording, mitochondrial studies, and ISG studies.	Used in Bulk-RNAseq and transplantation	Bulk-RNAseq, scRNAseq and transplantation	Used in Bulk RNAseq	Used in Bulk-RNAseq, transplantation	For Ca2+ recording, mitochondrial studies, and ISG studies.	For Ca2+ recording, mitochondrial studies, and ISG studies.	Used in transplantation, mitochondria/insulin staining.

[a]If you have used more than eight islet preparations, please complete additional forms as necessary
[b]For example, IIDP, ECIT, Alberta IsletCore
[c]Please specify the therapy/therapies
[d]Time of islet culture at the isolation centre, during shipment and at the receiving laboratory
[e]Please specify the test and the results
